# Water Insecurity, Social Perspectives, and Health Impacts in Private Drinking Water Sources in Pennsylvania: Two Systematic Literature Reviews

**DOI:** 10.1002/wat2.70049

**Published:** 2025-12-25

**Authors:** Lauren M. T. Broyles, Leslie B. Ford, Kaitlyn Barnhart, Siddhi Deshpande, Madeleine Todd, Erica Morse, Susan Boser, Lara B. Fowler, Andrew Warner, Asher Y. Rosinger

**Affiliations:** 1 Department of Biobehavioral Health, Pennsylvania State University, University Park, Pennsylvania, USA |; 2 Population Research Institute, Pennsylvania State University, University Park, Pennsylvania, USA |; 3 University of Michigan Medical School, Ann Arbor, Michigan, USA |; 4 Penn State Extension, College of Agricultural Sciences, Penn State University, University Park, Pennsylvania, USA |; 5 Penn State Sustainability, Pennsylvania State University, University Park, Pennsylvania, USA |; 6 Department of Ecosystem Science and Management, Pennsylvania State University, University Park, Pennsylvania, USA |; 7 Department of Anthropology, Pennsylvania State University, University Park, Pennsylvania, USA

**Keywords:** health, SDG 6, water insecurity, water perceptions, water quality

## Abstract

Over 43 million people in the United States rely on private drinking water supplies for domestic purposes, yet 20% of water wells are potentially contaminated in some form. In Pennsylvania, 2–3 million rural residents use private water supplies for domestic purposes, yet Pennsylvania has no state-wide well drilling standards or water quality testing requirements for private water sources. The lack of implemented standards and monitoring hides potential water supply and quality issues and may affect water trust, health, and overall water insecurity. Therefore, we conducted two interlinked systematic reviews to understand (1) social perspectives around water insecurity among private water supply users in Pennsylvania and (2) associations between private water sources and health outcomes. Using Scopus, Web of Science, PubMed, ProQuest, Compendex, and Google Scholar, we identified 10 articles (*n*1) for the first review and 15 for the second (*n*2). Results indicated water insecurity relates to perceived and measured water quality problems. Common sources of water quality issues were chemical contamination (*n*1 = 5, *n*2 = 8), biological contamination (*n*1 = 3, *n*2 = 7), and proximity to shale gas drilling (*n*1 = 6, *n*2 = 4). Four articles measured health effects including thyroid health, risk for Parkinson’s disease, and effects related to TCE/PCB contamination and proximity to shale gas drilling, while others discussed poor water quality potentially causing gastrointestinal illnesses. These reviews indicate opportunities for increased water testing for private water users and education for water quality health risks. Emerging literature emphasizes a surge of research interest in examining the social perceptions and health effects of water in Pennsylvania.

This article is categorized under:

Engineering Water > Water, Health, and Sanitation
Human Water > Water Governance
Science of Water > Water Quality

## 1 | Introduction

Water is critical for health (Rosinger and Young 2020), yet roughly two million people in the United States lack access to water for basic needs (Roller et al. 2019). The problem is larger still as closer to 60 million Americans do not drink their tap water due to distrust (Rosinger 2022; Rosinger et al. 2023). Water source avoidance due to uncertainty and health consequences associated with unsafe or inadequate supply can lead to water insecurity, or the inability to access and gain adequate, reliable, and safe water for well-being and a healthy lifestyle (Jepson et al. 2017). Thus, water insecurity is an emerging concern in the United States (Gaber et al. 2021; Meehan, Jepson, et al. 2020; Meehan, Jurjevich, et al. 2020; Nwanaji-Enwerem and Casey 2022; Rosinger 2022; Rosinger and Young 2024).

Yet, few states in the United States have been systematically evaluated. To date, water problems and water insecurity have been identified as an issue in several states, such as North Carolina (Hayes et al. 2025; Pieper et al. 2018; Workman and Shah 2023), Texas (Jepson 2014; Wutich et al. 2022), Virginia (Pieper et al. 2015), Alaska (Eichelberger 2018, 2019, 2010), Kentucky (Wies et al. 2020), Ohio (Toland et al. 2025), and nation-wide (Deitz and Meehan 2019; Hernandez and Pierce 2023; Javidi and Pierce 2018; Meehan, Jurjevich, et al. 2020), to name a few. State-level assessments are critical as laws related to drinking water governance vary from state to state. However, the Commonwealth of Pennsylvania, which has approximately 2.7 million rural Pennsylvania residents (nearly a quarter of the population) who use a private water supply (well, spring, or cistern), has yet to be assessed. Further, Pennsylvania is one of few states without statewide regulations on the location, construction, testing, or treatment of private drinking water supplies (Johnson et al. 2019; Swistock et al. 2015). Thus, there is a clear need for more research on experiences surrounding water insecurity in Pennsylvania.

Relying on voluntary, private management of domestic water systems creates numerous problems; most importantly are potential exposures to health-related pollutants with or without users’ knowledge. Further, private wells are not regulated by the EPA’s Safe Drinking Water Act (1974) and thus water quality is not monitored by the state, but rather is on the onus of the homeowners. Widespread contamination of private wells in Pennsylvania was first reported in a national study of 2600 homes in the late 1970s (Francis et al. 1982). Since then, mounting evidence has consistently shown that more than 40% of samples from private water systems in the Commonwealth fail at least one health-based drinking water standard—the most common include standards related to coliform bacteria, *Escherichia coli* bacteria, lead, nitrate, arsenic, and chemicals from various personal care products (Swistock et al. 2013; Kibuye et al. 2019). Residents with private or small, semipublic wells are considered the most vulnerable to waterborne illnesses (Craun 1986) as disease-causing bacteria have been found in private wells in Pennsylvania and linked to the proximity of septic systems (Lindsey et al. 2002; Murphy et al. 2020; Swistock et al. 2004). Despite substantial evidence of contamination, there has been little work done on water insecurity concerns and associated health impacts in Pennsylvania.

Further, Pennsylvania encompasses a variety of water issues. Water quality concerns have generally been related to extractive, industrial, urban and agricultural activities, including contaminants of emerging concern. These concerns increased following the hydraulic fracturing boom of the Marcellus shale formation for natural gas development, which expanded rapidly in 2008 (Muehlenbachs et al. 2015). The Marcellus shale formation is one of the largest natural gas deposits in the United States and covers more than half of Pennsylvania. While the Marcellus shale formation represents opportunity for increased economic activity, increased hydraulic fracturing of the Marcellus shale for gas development has led to localized impacts to groundwater and increased concern about private well water quality. There have been several health studies showing some level of correlation between health effects (e.g., higher risk of low birth weight and mental health impacts) and proximity to shale energy development in Pennsylvania (Casey et al. 2016; Currie et al. 2017; Hirsch et al. 2018); however, no studies have demonstrated direct exposure pathways, such as via private well water. Consequently, it is likely that households on well water living close to fracking sites are more likely to experience higher levels of water insecurity, yet there is no evidence on this yet. More broadly, there is limited understanding of perceptions of water insecurity risks and health outcomes among households in Pennsylvania that rely on private water supplies and how people cope with these issues.

To address these gaps, we conducted two linked systematic literature reviews focused on Pennsylvania as a case study to understand:

**RQ1**. *What are the social perspectives around water insecurity among those dependent on private water supplies in Pennsylvania?*
**RQ2**. *What are the human health outcomes related to private water sources in Pennsylvania? These reviews had a secondary aim of understanding the associated coping strategies of how people respond to these water problems*.

## 2 | Data and Methods

### 2.1 | Search Strategy

To conduct the two systematic reviews, we used the PRISMA systematic review guidelines (Page et al. 2021), where search terms were enlisted to retrieve articles from the following databases: Web of Science (Topic search), Compendex (Subject/Title search), ProQuest (including Psychinfo) (Abstract search), PubMed (Title/Abstract search), Scopus (including Science Direct) (Title/Abstract/Keywords search), SSRN (PA & Water Only) (Abstract search), PAIS (Abstract search), Google Scholar (first 200 peer-reviewed articles). To address RQ1, search terms included geographic criteria such as “Pennsylvania” or the name of a major catchment or watershed, “water” or other possible private water source terms, dimensions of water insecurity, dimensions of mental health and perceptions of water, beliefs and views on the water situation, and study population or scale of the study (Table 1). To address RQ2, we used search terms that included geographic criteria such as “Pennsylvania” or the name of a major catchment or watershed, “water” or other possible private water source terms, dimensions of water insecurity, health outcomes or categories, types of water contamination, and study population or scale of the study.

### 2.2 | Eligibility Criteria and Assessment of Bias

Based on both searches, we retained studies that met the following inclusion criteria: (a) published between 1970 and 2020; (b) discuss a private water source such as private wells, springs, cisterns, or groundwater; (c) include data specific to Pennsylvania; (d) written in English; (e) peer-reviewed journal article or government document; and (f) address either social perspectives of water or human health outcomes. The timeframe to include articles beginning in 1970 was chosen because it encompasses many water milestones in the United States and Pennsylvania, including the Clean Water Act of 1972 (The Clean Water Act 1972), the establishment of the Pennsylvania State Water Plan in 1972. The last search was conducted August 12, 2020.

### 2.3 | Screening Process

**FIGURE 1 |:**
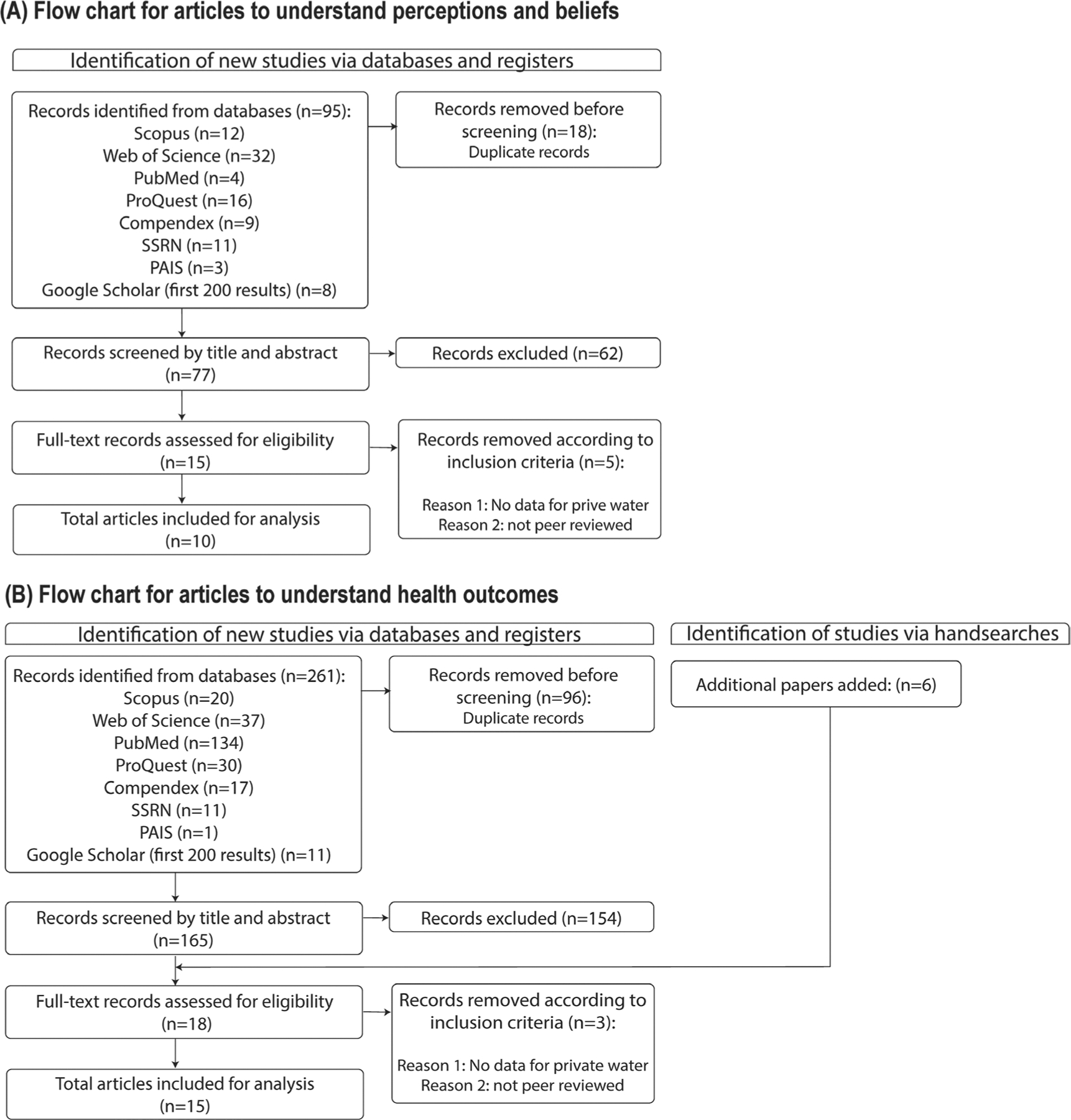
Flowchart of article screening and selection process to address research questions. Panel A describes the flowchart to address RQ1 related to perceptions and beliefs and Panel B describes RQ2 related to health effects of private drinking water sources in Pennsylvania. Adapted from Page et al. (2021).

Articles were exported to Endnote for a multi-step screening process (Figure 1). To address RQ1, a total of 95 articles were identified from the database search (Figure 1A). From these, 18 duplicates were removed prior to the screening process. All titles, abstracts, and full texts were screened by L.B.F., S.D., and K.B. Discrepancies were discussed until a consensus was reached. Full-text screening (*n* = 15) was completed independently by L.B.F., S.D., and K.B. After all screening iterations, the authors retained 10 articles.

To address RQ2, a total of 261 articles were identified from the database search (Figure 1B). From these, 96 duplicates were removed prior to screening according to exclusion criteria. An additional six articles identified through reviewing article references and hand searching via google scholar were added to the full-text review. After following the same methods as described above, the authors retained 15 articles.

### 2.4 | Data Collection and Synthesis Methods

Study characteristics from each paper were collected according to study methods (e.g., quantitative, qualitative, mixed methods), geographic location, setting (e.g., rural, urban, peri-urban), study population, water source type, and water uses mentioned (Tables S1 and S2). Methods were evaluated according to dates and duration of data collection, study type (e.g., longitudinal, cross-sectional, etc.), theory or framework, and study design. Water insecurity was evaluated according to water source, water acquisition type, water insecurity type, water insecurity causes, water insecurity timescale, and water related coping strategies (i.e., responses or adaptations to manage experiences of water insecurity (Venkataramanan et al. 2020)). Social perspectives were evaluated according to discussion of participant distrust surrounding their water source, preference for their water over public water, mental health, and organoleptic qualities (e.g., taste, smell, or color of water), beliefs and views. Health outcomes were evaluated according to general health risk assessment, specific conditions, specific causes, and organoleptic qualities. We evaluated both social perspective and health outcomes according to organoleptic qualities as they were found in articles from both reviews.

During peer-review, we conducted a final hand-search of Google Scholar in September 2025 to identify articles related to these research questions published since the last systematic search. We entered the keyword searches for both research questions into Google Scholar, limited the timeframe to articles published since 2021, and downloaded the first 50 articles that appeared in each search. These articles were then screened according to our inclusion/exclusion criteria, leaving 12 additional articles representative of emerging issues. We use this hand-search to supplement the primary analysis to examine how themes identified in our literature review have evolved. These hand-searches were not included in the primary analysis of the systematic literature review and are not reflected in Figure 1.

## 3 | Results

**FIGURE 2 |:**
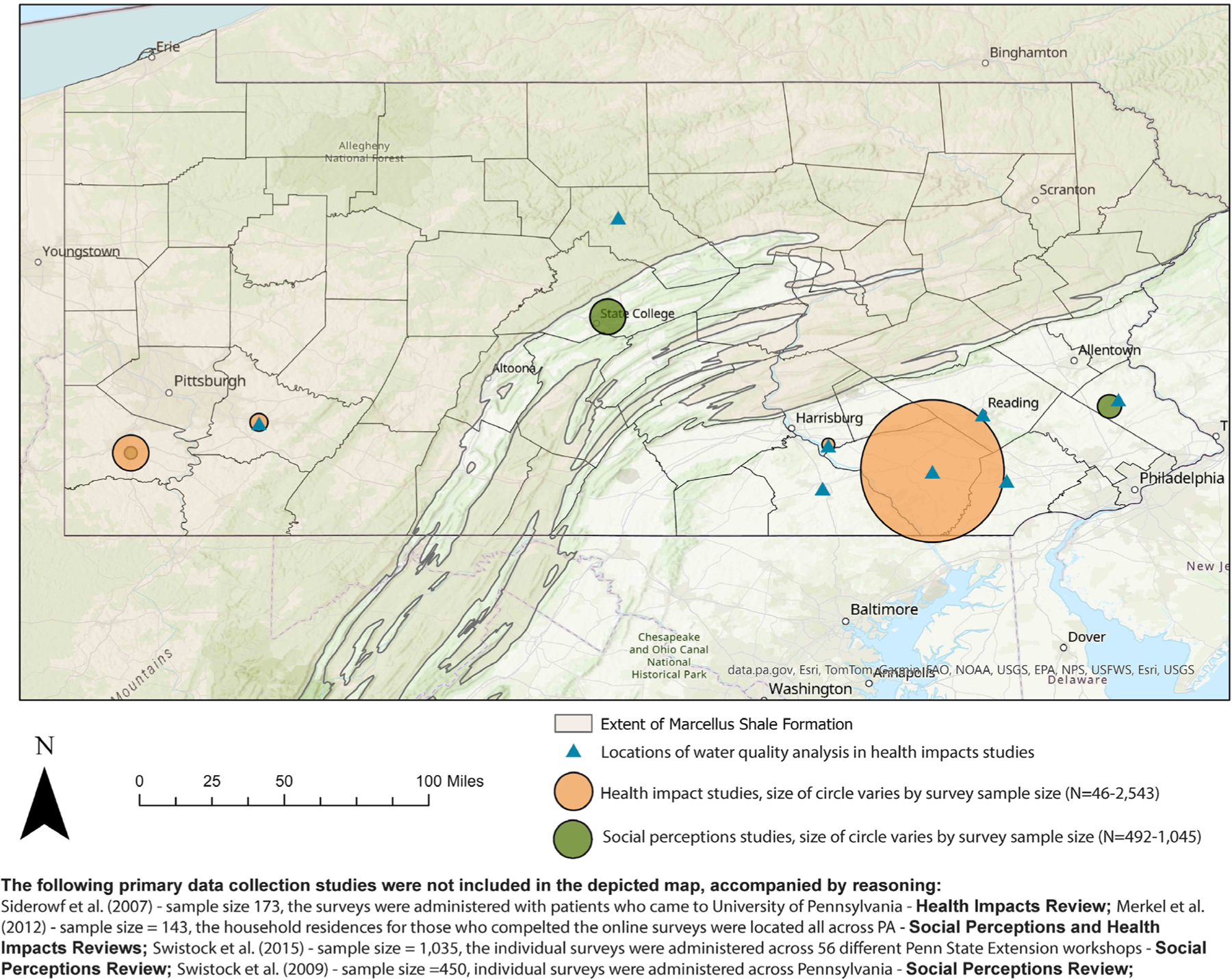
Map of approximate study locations identified from articles addressing social perceptions about private drinking water sources and those describing health outcomes in Pennsylvania.

In total, 10 articles were identified as dealing with social perceptions surrounding private drinking water sources in Pennsylvania (RQ1), and separately, 15 were related to health outcomes (RQ2), with 3 articles that overlapped between the two research questions. Among articles that addressed RQ1, one was a legal briefing and 9 were published in peer-reviewed literature, where 6 used primary data collection of individual or household responses (sample size, *N* = 143–1045), and 3 used large secondary datasets (*N* = 3646–229,946). Among articles for RQ2, 2 were United States Geological Survey (USGS) reports and 13 were peer-reviewed articles, where 6 dealt with primary household or individual data collection (*N* = 46–2543) and 1 used secondary household data (*N* = 229,946). Seven (five peer-reviewed and the two USGS reports) only collected water quality data. Figure 2 shows approximate locations of the primary household or individual survey and water quality data collection among articles across the two systematic reviews. The locations of study sites that collected data across a county or township were assigned to the county seat or the major city in the township. Given the vague locations described in some articles, these locations were randomly assigned to be representative of the described location (e.g., Southwest Pennsylvania). We do not show the locations of the articles that used secondary data because of large sample size and data which spanned tens of counties.

### 3.1 | Water Insecurity Across Pennsylvania

Water sources and water acquisition type (e.g., piped water, hauling water, fetching from springs, purchasing water) varied by paper with many mentioning multiple types of water provisioning, the most prevalent being private water wells. Of the 10 papers from RQ1, 60% (*n* = 6) discussed private water wells (Wrenn et al. 2016; Gopalakrishnan and Klaiber 2014; Merkel et al. 2012; Muehlenbachs et al. 2015; Rabinowitz et al. 2015; Swistock et al. 2009); 40% (*n* = 4) focused primarily on municipal or community water systems, with private water systems mentioned as a small percentage of the sample or as alternative water sources (Abdalla 1990; Abdalla et al. 1992; Gopalakrishnan and Klaiber 2014; Merkel et al. 2012); 20% (*n* = 2) focused on groundwater and surface water contamination broadly (Wrenn et al. 2016; Centner 2013); and 4 discussed springs (Abdalla 1990; Rabinowitz et al. 2015; Swistock et al. 2009, 2015). Of the 15 papers from RQ2, all discussed private water wells (100%, *n* = 15), of which two also mentioned springs (Kibuye et al. 2019; Rabinowitz et al. 2015), and one bottled water (Merkel et al. 2012).

**FIGURE 3 |:**
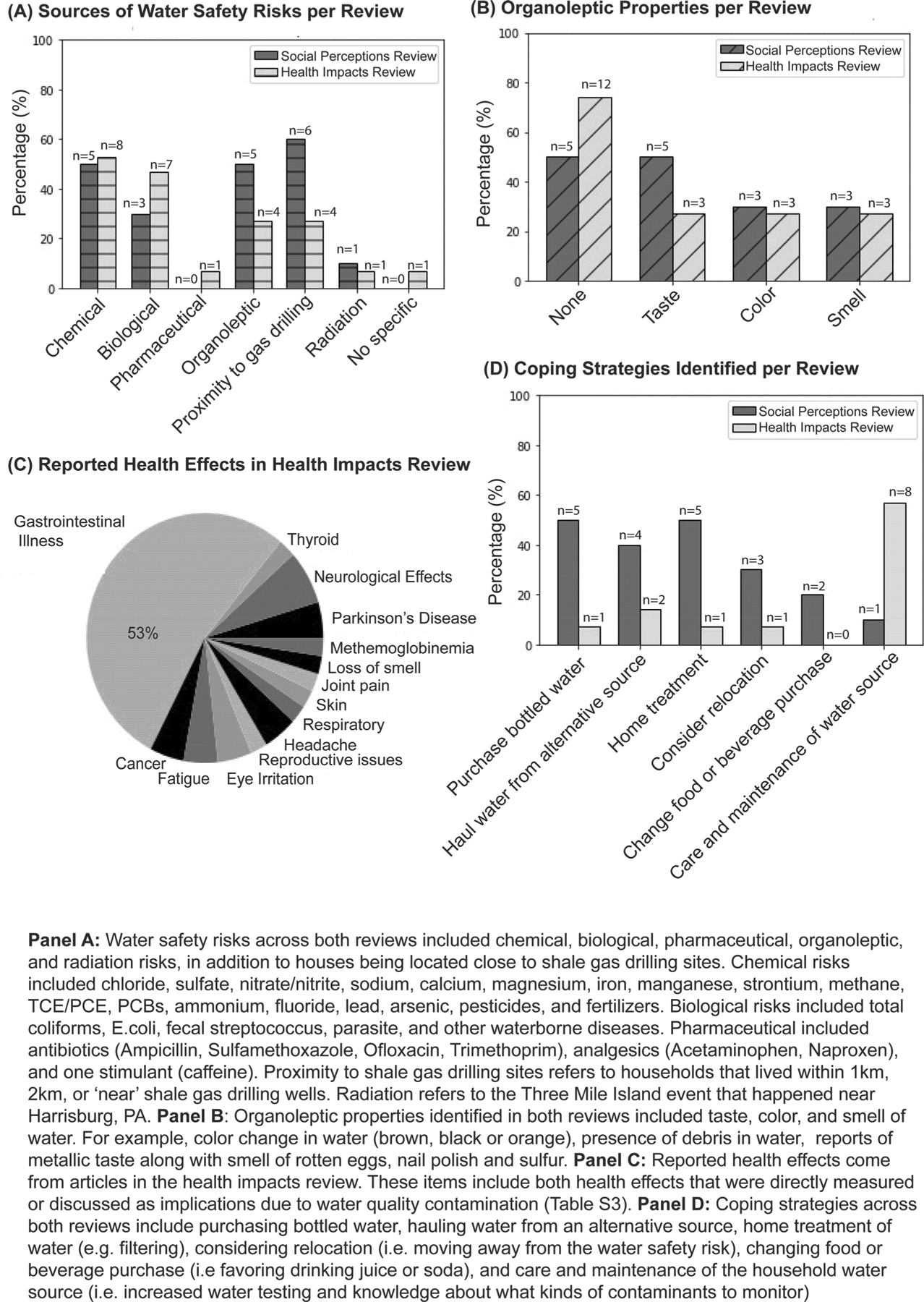
Summary results from the systematic reviews identifying: (A) water safety risks; (B) organoleptic properties; (C) health effects reported in articles about health impacts of private drinking water sources; and (D) coping strategies used.

Almost all articles from RQ1 dealt with some aspect of real or perceived water safety, as it was related to water quality (Figure 3). Water safety risks were both regionally based (i.e., where the risk was located) and dependent on the type of household water source. Most of these (*n* = 6, 60%) discussed risks related to proximity to shale gas drilling on local water supply as evidenced through property values and perceived or reported health impacts (Centner 2013; Gopalakrishnan and Klaiber 2014; Merkel et al. 2012; Muehlenbachs et al. 2015; Rabinowitz et al. 2015; Wrenn et al. 2016) (Figure 3A). For example, studies on perceived risk of shale gas drilling on drinking water were concentrated in the Marcellus Shale region, where many gas wells existed near homes with private groundwater drinking sources (Centner 2013; Gopalakrishnan and Klaiber 2014), while biological contamination in drinking water sources was focused on private wells and natural spring use in the northwestern part of PA where natural spring usage is high (Swistock et al. 2009, 2015).

Most dealt with only perceived risk (*n* = 8, 80%), while two included objective water quality measurements (Swistock et al. 2009, 2013), and two dealt with contamination events (Abdalla 1990; Abdalla et al. 1992). Thirty percent (*n* = 3) considered groundwater contamination of volatile organic compounds, such as trichloroethylene (TCE) and tetrachloroethylene (PCE), and radiation contamination (Abdalla 1990; Abdalla et al. 1992; Merkel et al. 2012). Wrenn et al. (2016) identified chemicals leaching from shale gas drilling as a concern. Swistock et al. (2009) tested for lead, nitrates, arsenic, and pesticides in household drinking water.

Organoleptic properties were important in articles from both reviews as organoleptic properties and perceptions influenced behavior (Figure 3A,B). Five articles from RQ1 (50%) discussed organoleptic properties (Abdalla et al. 1992; Merkel et al. 2012; Rabinowitz et al. 2015; Swistock et al. 2009, 2015) while four articles from RQ2 (27%) did so (Alawattegama et al. 2015; McDernnott-Levy and Kaktins 2012); two overlapped with social perceptions review (Merkel et al. 2012; Rabinowitz et al. 2015) (Figure 3A,B). Taste was identified as a reason people purchased bottled water (Abdalla et al. 1992), installed water treatment in the home (Swistock et al. 2009), and used roadside springs, as they claimed them to be natural and good tasting (Swistock et al. 2015). Others reported general dissatisfaction with water taste, smell, and appearance in areas that had shale gas drilling and others across the state (Alawattegama et al. 2015; McDernnott-Levy and Kaktins 2012; Merkel et al. 2012; Rabinowitz et al. 2015).

Real or perceived water safety issues related to water quality from RQ2 centered on biological contamination, proximity to shale gas extraction, and other contaminants (Table S3). Forty percent (*n* = 6) measured biological contamination of water (Alawattegama et al. 2015; Bickford et al. 1996; Lindsey et al. 2002; Murphy et al. 2020; Swistock et al. 2013; Swistock and Sharpe 2005), with one discussing health effects without measured water quality (Muehlenbachs et al. 2015) (Figure 3A). Twenty-seven percent (*n* = 4) measured water quality or perceived risk related to proximity to shale gas extraction (Alawattegama et al. 2015; McDernnott-Levy and Kaktins 2012; Muehlenbachs et al. 2015; Rabinowitz et al. 2015). Twenty percent (*n* = 3) assessed nitrate contamination (Aschebrook-Kilfoy et al. 2012; Swistock et al. 2013; White et al. 2013); 7% (*n* = 1) measured TCE contamination (Logue et al. 1985); 7% (*n* = 1) related to chemicals from shale gas extraction (Alawattegama et al. 2015), and 7% (*n* = 1) pharmaceuticals (Kibuye et al. 2019). Swistock et al. (2013) measured drinking water pH, triazine pesticides, total coliforms, *E. coli* bacteria, nitrates, arsenic, and lead concentration. McDernnott-Levy and Kaktins (2012) did not measure water quality directly but discussed health impacts of groundwater and surface water contaminants related to shale drilling including the following: methane, benzene, xylenes, purgeable hydrocarbons, bromide, and gasoline and diesel byproducts.

Water quantity and access were discussed among papers in both reviews to a lesser degree than water safety/quality. In the social perspectives review (RQ1), only two articles addressed water quantity and access (Swistock et al. 2009, 2013). Six discussed proxies of accessibility, such as cost of alternative sources and coping strategies (Abdalla 1990; Abdalla et al. 1992; Wrenn et al. 2016; Merkel et al. 2012; Muehlenbachs et al. 2015; Swistock et al. 2009). In the health impacts review, only one paper mentioned the quantity of water being an issue (Alawattegama et al. 2015).

### 3.2 | Social Perceptions

The themes of trust and distrust were described in all articles from RQ1, where distrust often led to residents trusting and relying on their private water sources to a greater extent than municipal supply. In work by Abdalla (1990), which documented public response to TCE contamination of drinking water in College Township, PA, residents indicated frustration over the lack of safety standards and differing opinions of the health risks of the water supply. As a result, residents trusted spring sources more than piped water sources. In the Borough of Perkasie, PA, chemical contamination led to distrust of the community water supply and the belief that hauled water is better/safer than tap water (Abdalla et al. 1992). Sixty percent (*n* = 6) mentioned water quality linked to shale gas wells or fracking (Centner 2013; Wrenn et al. 2016; Gopalakrishnan and Klaiber 2014; Merkel et al. 2012; Muehlenbachs et al. 2015; Rabinowitz et al. 2015). Merkel et al. (2012) discussed parents’ perceptions about drinking water safety for their children and risks related to fracking, radiation, and general tap water distrust. Memory of the historical Three Mile Island nuclear accident caused residents to worry over nearby drinking water supplies (Merkel et al. 2012). For some, distrust of the water sources extended to the insurability and value of properties near natural gas drilling (Gopalakrishnan and Klaiber 2014; Muehlenbachs et al. 2015). There was higher trust in roadside springs water because it was perceived as natural and having better taste (Swistock et al. 2015). This is an area for future research given the high use of roadside springs (~10% of Pennsylvanians) for drinking water coupled with their lack of regulation and dearth of monitoring.

### 3.3 | Human Health Impacts

Of the RQ2 articles, only four measured health effects in their study population, while the remaining discussed health impacts in relation to measured contaminants (Table S3). Measured health impacts include thyroid stimulating hormone in an Amish population, where the association was found between modeled nitrate in groundwater and clinical hyperthyroidism, clinical hypothyroidism, subclinical hyperthyroidism, subclinical hypothyroidism (Aschebrook-Kilfoy et al. 2012) (Figure 3C, Table S3). Logue et al. (1985) reported increased fatigue, eye irritation, and diarrhea in a community exposed to TCE and PCBs in groundwater. Similarly, in households near shale gas wells, Rabinowitz et al. (2015) reported respiratory, skin, neurological, and gastrointestinal issues, including throat and nasal irritation, eye burning, sinus problems, headaches, skin problems, loss of smell, cough, nosebleeds, and painful joints. Finally, Siderowf et al. (2007) found that well water use was associated with impaired olfactory function in relatives of Parkinson’s disease patients, an indicator connected to later Parkinson’s disease onset. Other reported but unmeasured health concerns can be found in Table S3.

### 3.4 | Coping Strategies

Coping strategies differed among the two reviews (Figure 3D). In response to perceptions and beliefs, people most frequently sought to cope through the purchase of bottled water (*n* = 5, 50%), hauling water from alternative sources (*n* = 4, 40%), treating their water at home (*n* = 5, 50%), considering relocation (*n* = 3, 30%), and changing their food or beverage purchases (*n* = 2, 20%) (Abdalla 1990; Abdalla et al. 1992; Gopalakrishnan and Klaiber 2014; Merkel et al. 2012; Muehlenbachs et al. 2015; Swistock et al. 2009, 2015; Wrenn et al. 2016). In contrast, health impacts of drinking water articles emphasized testing, maintenance, and care of water sources (*n* = 8, 53.3%).

### 3.5 | Emerging Social Perception and Health Impacts Water Insecurity Issues in PA

Since 2020, there has been a surge of research evaluating social perceptions and health impacts related to drinking water issues in the United States broadly and in Pennsylvania specifically. In PA, water insecurity perceptions related to concerns about the effects of shale gas drilling (Clark et al. 2021, 2022; Hill and Ma 2022; Shaheen et al. 2024), per- and poly-fluoroalkyl substances (PFAS) (Breitmeyer et al. 2023; Jones et al. 2025; Kosiarski et al. 2025), historic coal mining (Bram et al. 2024), road deicing (Cruz et al. 2022), drinking water violations broadly (DiSalvo and Hill 2024), waterborne disease outbreaks (Kunz et al. 2024), and the effects of power plant discharges on downstream populations (Good and VanBriesen 2017). The range of analyses in this literature included general concerns about the effects of environmental risks on drinking water through measured water quality, exposures, and health outcomes. Notable health impacts work included the following: testing associations between PFAS-contaminated water and cancer incidence (Jones et al. 2025); increased incidence of preterm birth and low birth weight due to in utero exposure to shale gas development (Hill and Ma 2022) and drinking water violations (DiSalvo and Hill 2024); and increased groundwater salinization due to shale gas (Shaheen et al. 2024) and road deicing (Cruz et al. 2022).

## 4 | Discussion

This paper aimed to better understand the state of research on water insecurity in Pennsylvania related to social perspectives and health outcomes among private water source users, with a secondary aim of understanding coping strategies. We found that overall, few studies addressed these questions. No article assessed actual water insecurity experiences but dealt with a limited number of dimensions of water insecurity including quality, quantity, and access. As this is among the first articles to take stock of water insecurity in Pennsylvania, we highlight important areas to begin addressing water security throughout the Commonwealth, while we also contribute to the broader dialog of water insecurity in the United States (Deitz and Meehan 2019; Eichelberger 2018, 2019; Eichelberger 2010; Hayes et al. 2025; Hernandez and Pierce 2023; Javidi and Pierce 2018; Jepson 2014; Meehan, Jepson, et al. 2020; Meehan, Jurjevich, et al. 2020; Pieper et al. 2018; Wies et al. 2020; Workman and Shah 2023; Wutich et al. 2022). This review contributes important evidence that will be further expanded upon with ongoing water insecurity work in Pennsylvania, which is a research site for the validation of scale to address and measure water insecurity in the United States (Pearson et al. 2025).

Addressing RQ1, articles from the first review discussed social perceptions among water users in a few ways. First, the review revealed results consistent with broader US literature that there is higher reliance on bottled water where there is mistrust in a water source, whether that is tap or private water (Meehan, Jepson, et al. 2020; Rosinger 2022; Rosinger et al. 2022; Rosinger and Young 2024). Second, we found some evidence that private water source users may trust their water sources more than public water, despite the risk for contamination. Third, trust of private water sources varied by type. Interestingly, there was greater trust in spring water, whereas articles that analyzed private well usage more often discussed contamination concerns (e.g., proximity to shale gas drilling and drilling byproducts leaking into groundwater supply). Perceptions of risks are regionally based depending on where the threat to water safety is located. It is worth noting, however, that these perceptions seem to be skewed toward new and/or geographically discrete activities (e.g., individual fracking wells), while perhaps down-playing long-standing activities (e.g., agriculture) even though actual monitoring (Swistock et al. 2009, 2013; Swistock and Sharpe 2005) has demonstrated these are the most documented sources of contaminants detected in private drinking water wells. Given the few, regionally biased, and mixed results related to trust and distrust of private water sources and perceived risks, there is need for increased water quality testing for private water users in Pennsylvania. This is especially important as widespread occurrence of PFAS in private wells in Pennsylvania is increasingly a concern to drinking water safety (Kosiarski et al. 2025), and households with aged plumbing infrastructure may be at risk of waterborne lead exposure (Pieper et al. 2015, 2018). Overall, our findings emphasize the same themes of an emerging literature that shows private water users in the United States are a vulnerable population to water insecurity and unaccounted health risks (Long et al. 2025; Meehan, Jepson, et al. 2020; Pieper et al. 2015, 2018; Wutich et al. 2022).

Addressing RQ2, we found few studies measured health effects related to private water sources. This is an area that deserves much greater attention due to the wide array of health effects that could be caused by exposure to water quality contamination. We only found four articles with empirical measurements of health effects (Aschebrook-Kilfoy et al. 2012; Logue et al. 1985; Rabinowitz et al. 2015; Siderowf et al. 2007). Studies that measured water quality only discussed *potential* health effects related to the water quality results (e.g., gastrointestinal illness and waterborne diseases) (Bickford et al. 1996; Lindsey et al. 2002; Murphy et al. 2020; Swistock et al. 2013; Swistock and Sharpe 2005). Thus, there is a need for increased evidence that directly connects water quality to downstream health effects.

Emerging literature emphasized the need for greater understanding of environmental risks on drinking water and health effects. Important findings will come from the work by Jones et al. (2025) as they investigate the associations between PFAS and cancer incidence. Further, increased salinization of drinking water due to road deicing (Cruz et al. 2022) and groundwater due to shale gas development (Shaheen et al. 2024) is an important public health issue which has been overlooked in the United States Increased salinization of drinking water sources may lead to increased risk of hypertension and kidney problems, especially for those on restricted sodium diets; yet there are no current health based standards by the Environmental Protection Agency for drinking water sodium (Rosinger et al. 2025). Work by DiSalvo and Hill (2024) and Hill and Ma (2022) alert us to the negative consequences of poor water quality on maternal and infant health and well-being.

As such, there is a critical need for water testing education and drinking water health literacy. The Master Well Owner Network (https://extension.psu.edu/programs/mwon) can provide well owners with resources and education for water testing regarding frequency, information on state-certified laboratories that perform testing, and water quality parameters suggested for private wells in shale drilling areas (MWON 2025; Swistock et al. 2009). Medical and public health professionals can also help communicate drinking water risks, appropriate mitigation strategies, and education on the meaning of water quality reports and how to access those on the Pennsylvania Department of Environmental Protection (DEP) website (McDernnott-Levy and Kaktins 2012).

Understanding drinking water risks is especially important for parents of young children. Merkel et al. (2012) found more than half of the parents in their study would like advice on drinking water quality from their child’s pediatrician. In addition to general water quality risks, information about whether water sources are fluoridated and supplemental resources is crucial as fluoride is important for healthy teeth development particularly during critical child growth years (Prasad et al. 2021). Not all public water sources are fluoridated in Pennsylvania or even the United States more broadly (Hung et al. 2023; Prasad et al. 2021). Lack of fluoride in drinking water may be a larger issue among private water users, whose water sources are unregulated. Recent research underscores the need for parents of young children to understand drinking water risks as unhealthy drinking water could have negative birth outcomes (DiSalvo and Hill 2024; Hill and Ma 2022).

Increased water testing may also reduce household coping strategies and averting expenditures. For example, in the case that water quality test results indicate safer water than previously perceived, households may stop purchasing bottled water. Bottled water purchases in Pennsylvania counties with shale drilling activities were estimated to accumulate to $12.9 million in 2009 and $19 million in 2010 (Wrenn et al. 2016). While outside the scope of our two systematic reviews, future work should disentangle the role of wastewater and private ownership of septic tanks as these are intertwined with private well ownership and groundwater quality. Evidence from southeastern Pennsylvania demonstrated that contamination of private wells is most likely caused by septic system leakage, which can lead to gastrointestinal illnesses (Murphy et al. 2020). Close to 10% of the US population owns both private wells and septic tanks, with a higher percentage (12.6%) of the Northeast relying on both (Hernandez and Pierce 2023). This population faces a higher burden of health and reliability issues than those who use public water and sewer services, which further emphasizes the need for increased drinking water testing.

## 5 | Conclusion

In this work, we conducted two systematic literature reviews to understand the social perspectives and health outcomes surrounding water insecurity among those dependent on private water sources in Pennsylvania and related coping strategies. There are many literature gaps surrounding these two important aspects of household water provisioning in Pennsylvania. Household water insecurity was mostly assessed regarding perceived and measured water safety, with only one article addressing water quantity and affordability. Most articles about social impacts discussed risks from proximity to shale gas drilling on local water supply as evidenced through property values and perceived or reported health impacts. Of the articles about human health outcomes, only four measured health effects among study participants, while other articles discussed health concerns related to water quality contamination. Coping strategies included purchasing bottled water, home treatment of drinking water, hauling water from alternative sources, considering relocation, changing food or beverage purchases, and care and maintenance of the household water source. Emerging literature demonstrated an increased amount of interest and research in the several themes identified in our systematic reviews, particularly, for those on unregulated private water sources. This review also highlights the need for and implementation of a validated tool measuring water insecurity experiences in the U.S. to understand the magnitude of water insecurity in places like Pennsylvania as well as to better understand how water insecurity affects health outcomes.

## Supplementary Material

Table S3

Table S2

Table S1

Additional supporting information can be found online in the Supporting Information section. **Table S1:** Study characteristics of studies related to social perspectives on water. **Table S2:** Study characteristics of studies related to health impacts of water. **Table S3:** Health effects per article.

## Figures and Tables

**TABLE 1 |:** Systematic review search terms for RQ1 and RQ2.

Search terms for RQ1	((Pennsylvania OR “Mid Atlantic catchment” OR “Mid Atlantic drainage basin” OR “eastern use watershed” OR “susquehanna watershed” OR “Potomac watershed” OR “Ohio Watershed” OR “Genesee Watershed” OR “Delaware watershed” OR “Erie watershed”) AND (water) AND (drinking AND water OR ground AND water OR groundwater OR private AND water OR well OR spring OR cistern OR underground AND water) AND (water AND access OR access OR water AND insecurity OR insecurity OR water AND availability OR availability OR water AND quality OR quality OR water AND resources OR resources OR water AND supply OR supply OR water AND scarcity OR scarcity OR water AND security OR security OR water AND stress OR stress OR water AND safety OR safety OR water AND reliability OR reliability) AND (beliefs OR perceptions OR distrust OR trust OR mental OR social OR psychological OR emotion OR coping OR cope OR adapt OR behavior OR strateg* OR manage* OR surviv* OR psychosocial OR anxiety OR views OR opinions OR concerns) AND (equity OR justice OR governance OR “Environmental Rights Amendment” OR poverty OR “Water Poverty Index” OR right OR “Human Right” OR “environmental injustice” OR risk OR afford* OR economic OR cost) AND (individual OR household OR home OR communit* OR compound* OR famil* OR parent OR child))
Search terms for RQ2	((Pennsylvania OR “Mid Atlantic catchment” OR “Mid Atlantic drainage basin” OR “Eastern US Watershed” OR “Susquehanna watershed” OR “Potomac watershed” OR “Ohio Watershed” OR “Genesee Watershed” OR “Delaware watershed” OR “Erie watershed”) AND (Water) AND (Drinking OR Ground OR groundwater OR Private OR Well OR Spring OR Cistern OR Underground) AND (health OR public health OR “birth defect” OR development OR obesity OR coliform OR diarrhea OR mental health OR reproduction OR Neurologic OR gastrointestinal OR safety OR disease OR purification OR purity OR illness OR sick* OR cancer) AND (Water Access OR access OR Water Insecurity OR Insecurity OR Water Availability OR Availability OR Water Quality OR Quality OR Water Resources OR Resources OR Water Supply OR Supply OR Water Scarcity OR Scarcity OR Water Security OR Security OR Water Stress OR stress OR water safety OR safety OR water reliability OR reliability) AND (water contamination OR contaminant* OR “waterborne pathogen” OR Herbicide OR Pesticide OR Agricultural OR “Heavy metal” OR lead OR industrial OR Radionuclide OR metal* OR pharmaceutical) AND (Individual OR Household OR Home OR Communit* OR Compound* OR famil* OR Parent OR Child))

## Data Availability

Data sharing is not applicable to this article as no new data were created or analyzed in this study.
