# Supplementary material for: Water Insecurity, Social Perspectives, and Health Impacts in Private Drinking Water Sources in Pennsylvania: Two Systematic Literature Reviews
Source: WIREs Water. Author manuscript; Available in PMC 2026 Apr 22. (PMC13099076; doi:10.1002/wat2.70049)
Supplement: Table S3 [file NIHMS2165101-supplement-Table_S3.docx]

| Table S3. Health effects per article | | | |  |
| --- | --- | --- | --- | --- |
| Authors | Contaminants of concern | Water quality sampling and analysis | Health effects | Were the health effects measured or merely discussed in context? |
| Alawattegama et al. (2015) | Proximity to shale gas extraction; Chloride, sulfate, nitrate, sodium, calcium, magnesium, iron, manganese and strontium were commonly found. High levels of manganese in some households. Methane, coliforms, and E. coli in a few households | Yes | Parkinson’s disease, lower IQ in children | Discussed |
| Aschebrook-Kilfoy et al. (2012) | Nitrate levels in well water supplies | No (used secondary data) | Thyroid stimulating hormone (measured); Association of nitrate with clinical hyperthyroidism, clinical hypothyroidism, sub-clinical hyperthyroidism, sub-clinical hypothyroidism | Measured |
| Bickford et al.  (1996) | Microbial contamination | Yes | Waterborne diseases | Discussed |
| Kibuye et al. (2019) | Pharmaceuticals, caffeine | Yes | Unknown effects of contaminants on health | Discussed |
| Lindsey et al. (2002) | Microbial contamination | Yes | Gastrointestinal issues, stomach cancer and other illnesses | Discussed |
| Logue et al. (1985) | TCEs, PCBs | Yes | Sleepiness, eye irritation and diarrhea among households that were exposed to contamination | Measured |
| McDernnott-Levy and Kaktins (2012) | Proximity to shale drilling; high concentrations of benzene, xylenes, purgeable hydrocarbons, gasoline and diesel byproducts | No | Chronic conditions associated with chemicals include neurotoxicity, reproductive problems, birth defects and cancer. More acute reports from people in the region include headaches, nose bleeds, nausea, fainting, stomach pain and fatigue | Discussed |
| Merkel et al. (2012) | Parental perception of water quality | No | Concerned about potential contamination of tap water by natural gas drilling, nuclear power plants, and parasites (gastrointestinal illness) from tap water | Discussed |
| Muehlenbachs et al. (2015) | Proximity to shale gas wells | No | Gastrointestinal illness, generally | Discussed |
| Murphy et al. (2020) | Microbial contamination | Yes | Gastrointestinal illness, generally | Discussed |
| Rabinowitz et al. (2015) | Proximity to shale gas wells | No | Respiratory, skin, neurological, and gastrointestinal. Other reported symptoms include throat and nasal irritation, eye burning, sinus problems, headaches, skin problems, loss of smell, cough, nosebleeds and painful joints | Measured |
| Siderowf et al. (2007) | Well-water use (for drinking, domestic needs) | No | Impaired olfactory function in relatives of Parkinson’s Disease patients (which is associated with later Parkinson’s Disease onset) | Measured |
| Swistock et al. (2005) | Total coliform and E.coli bacteria | Yes | Gastrointestinal illness, generally | Discussed |
| Swistock et al. (2013) | Total coliform, E. coli, lead, nitrate, arsenic, pesticides | Yes | Health-based standard violations | Discussed |
| White set al. (2013) | Nitrate in drinking water wells | Yes | Methemoglobinemia (Blue-baby syndrome) | Discussed |
