# Supplementary material for: Water Insecurity, Social Perspectives, and Health Impacts in Private Drinking Water Sources in Pennsylvania: Two Systematic Literature Reviews
Source: WIREs Water. Author manuscript; Available in PMC 2026 Apr 22. (PMC13099076; doi:10.1002/wat2.70049)
Supplement: Table S2 [file NIHMS2165101-supplement-Table_S2.docx]

| Table S2. Study Characteristics of Studies Related to Health Impacts of Water | | | | | | | | | | | |
| --- | --- | --- | --- | --- | --- | --- | --- | --- | --- | --- | --- |
| **Author (s)** | **Title** | **Manuscript Type** | **Publication Year** | **Location** | **Rural, Urban, Peri-urban** | **Study Population** | **Study Timeframe** | **Water sources** | **Water uses** | **Study design** |  |
| S. K. Alawattegama; T. Kondratyuk; R. Krynock; M. Bricker; J. K. Rutter; D. J. Bain; J. F. Stolz | Well water contamination in a rural community in southwestern Pennsylvania near unconventional shale gas extraction | Journal Article | 2015 | Southwestern PA community | Not specified but most likely rural or peri-urban | Community survey | Fall of 2011 to spring of 2014 | Private wells | "water needs", specifically drinking but most likely all other usual needs as well. | Mixed methods |  |
| B. Aschebrook-Kilfoy; S. L. Heltshe; J. R. Nuckols; M. M. Sabra; A. R. Shuldiner; B. D. Mitchell; M. Airola; T. R. Holford; Y. Zhang; M. H. Ward | Modeled nitrate levels in well water supplies and prevalence of abnormal thyroid conditions among the Old Order Amish in Pennsylvania | Journal Article | 2012 | Lancaster, Chester, and Lebanon counties | Rural because the population is mainly Amish | Individuals looked at for thyroid problems | nitrate levels measured from 1976-2006, TSH measured in the population from 1995-2008 | private well water | drinking water | Quantitative |  |
| Tammy M. Bickford, Bruce D. Lindsey, and M.R. Beaver | Bacteriological quality of ground water used for household supply, Lower Susquehanna River basin, Pennsylvania and Maryland | Report | 1996 | Lower Susquehanna river basin PA | All | household wells | 1993-1995 | private well | drinking | Quantitative |  |
| F. A. Kibuye; H. E. Gall; K. R. Elkin; B. Swistock; T. L. Veith; J. E. Watson; H. A. Elliott | Occurrence, Concentrations, and Risks of Pharmaceutical Compounds in Private Wells in Central Pennsylvania | Journal Article | 2019 | West branch of the Susquehanna river basin in central PA | Rural | homeowners using groundwater, either private wells or springs | samples taken throughout the winter of 2017, January-March | 24 private wells and 2 springs | sampled groundwater was used as primary drinking water in participating households | Quantitative for human health risk assessment |  |
| Bruce D. Lindsey, Jennifer S. Rasberry, and Tammy M. Zimmerman | Microbiological quality of water from noncommunity supply wells in carbonate and crystalline aquifers of Pennsylvania | Report | 2002 | All over PA, mostly southeast and central | All | community/household | september 2000 - January 2001 | wells | drinking and household use | Quantitative |  |
| J. N. Logue; R. M. Stroman; D. Reid; C. W. Hayes; K. Sivarajah | Investigation of potential health effects associated with well water chemical contamination in Londonderry Township, Pennsylvania, U.S.A | Journal Article | 1985 | Londonderry township, Dauphin county, PA | Rural/Peri-urban | households had their water tested, health survey completed by residents | water tested in July 1983, questionnaire administered in september 1983 | private well water | drinking water, and It can be inferred that it was also used for washing because of reported eye irritation | mixed methods |  |
| R. McDermott-Levy; N. Kaktins | Preserving health in the Marcellus region | Journal Article | 2012 | Marcellus shale region of Pennsylvania | Not specified but most likely rural | Community, paper discusses community health nurses educating communities about their water quality | Review about CHN's teaching communities in different instances of contamination throughout PA marcellus shale | private wells affected by marcellus shale drilling | drinking water, cooking, bathing | data not collected |  |
| L. Merkel; C. Bicking; D. Sekhar | Parents' perceptions of water safety and quality | Journal Article | 2012 | Online questionnaire all over Pennsylvania | All | Directed towards parents, so probably more family/household specific | A one time internet survey was completed in spring of 2011 | bottled water, public water, or private wells for drinking water consumption. | This study mainly focuses on drinking water, and if parents have discussed their water sources with their doctor, and how this has impacted child health | mixed methods |  |
| L. Muehlenbachs; E. Spiller; C. Timmins | The Housing Market Impacts of Shale Gas Development | Journal Article |  | Counties all over PA | Rural and Peri-urban | focused on households | data was analyzed that was collected between 1995 and 2012 | private wells | household (drinking) | quantitative |  |
| H. M. Murphy; S. McGinnis; R. Blunt; J. Stokdyk; J. W. Wu; A. Cagle; D. M. Denno; S. Spencer; A. Firnstahl; M. A. Borchardt | Septic Systems and Rainfall Influence Human Fecal Marker and Indicator Organism Occurrence in Private Wells in Southeastern Pennsylvania | Journal Article | 2020 | Bucks and Montgomery counties | Peri-urban | private wells supplying multiple households, community level? | water samples collected every other week for 2 months in 2016, and then recollected at 3 of the original wells in 2017 for 4 months weekly | private wells | household use, drinking | quantitative |  |
| Peter M Rabinowitz^1^, Ilya B Slizovskiy, Vanessa Lamers, Sally J Trufan, Theodore R Holford, James D Dziura, Peter N Peduzzi, Michael J Kane, John S Reif, Theresa R Weiss, Meredith H Stowe | Proximity to natural gas wells and reported health status: results of a household survey in Washington County, Pennsylvania | Journal Article | 2015 | Washington County, PA | Urban/Peri-urban | household survey | survey administered in summer 2012 | does not specify public or private water | mostly drinking mentioned, some mention of being exposed to contaminants through cooking, showering and swimming | quantitative |  |
| Andrew Siderowf 1, Danna Jennings, James Connolly, Richard L Doty, Kenneth Marek, Matthew B Stern | Risk factors for Parkinson's disease and impaired olfaction in relatives of patients with Parkinson's disease | Journal Article | 2007 | Participants came to University of Pennsylvania | All | individual questionnaire | 2007 | drinking from well water and association with PD | drinking | mixed methods |  |
| Swistock, Bryan R; Sharpe, William E | The Influence of Well Construction on Bacterial Contamination of Private Water Wells in Pennsylvania | Journal Article | 2005 | Wells all over PA | All | household | Feb-02 | private water wells | not specified but most likely household use | mixed methods |  |
| Bryan R. Swistock, Stephanie Clemens, William E. Sharpe and Shawn Rummel | Water Quality and Management of Private Drinking Water Wells in Pennsylvania | Journal Article | 2013 | Private wells across PA | All | household | 2006-2007 | private water wells | household use | Quantitative |  |
| White, P. Ruble, C. L., Lane, M. E. | The effect of changes in land use on nitrate concentration in water supply wells in southern Chester County PA | Journal Article | 2012 | Southern Chester County | Rural/Peri-Urban | household | Feb-02 | private water wells | not specified but most likely household use | Quantitative |  |
