# Supplementary material for: Water Insecurity, Social Perspectives, and Health Impacts in Private Drinking Water Sources in Pennsylvania: Two Systematic Literature Reviews
Source: WIREs Water. Author manuscript; Available in PMC 2026 Apr 22. (PMC13099076; doi:10.1002/wat2.70049)
Supplement: Table S1 [file NIHMS2165101-supplement-Table_S1.docx]

| Table S1. Study Characteristics of Studies Related to Social Perspectives on Water | | | | | | | | | | |
| --- | --- | --- | --- | --- | --- | --- | --- | --- | --- | --- |
| **Author (s)** | **Title** | **Manuscript Type** | **Publication Year** | **Location** | **Rural, Urban, Peri-urban** | **Study Population** | **Study Timeframe** | **Water sources** | **Water uses** | **Study design** |
| C. W. Abdalla | Measuring economic losses from  ground water contamination. An investigation of household avoidance costs | Journal Article | 1990 | College, Township | Peri-Urban | Individual on behalf of residence, household, community Study Population 1596 residential customers (~5000 ppl). Response rate 70% 1045/1498 surveys returned after two follow ups; 306 respondents hauled water | Late June 1987 Penn DER announced PCE contamination in Water Supply. Dec 1987 System discontinued use of Bathgate Spring. Questionnaire, there were two follow ups.  administered Feb 1988 | Municipal water users whose main water source is groundwater; alternative sources: springs or an uncontaminated source. | Used private water as an  alternative primary water supply. Paper mentions using primary supply for cooking, drinking. | Questionnaire Mix of quantitative and qualitative |
| C. W. Abdalla: B. A. Roach; D. J. Epp | Valuing Environmental Quality  Changes Using Averting Expenditures: an Application to Groundwater Contamination | Journal Article | 1992 | Borough of Perkasie, PA, located in Southeastern PA. | Peri-urban (Borough) | This study focuses on household/family level decisions within the Perkasie community  761 useable questionnaires were received. Response rate was 46.9%. 50 non-respondents were interviewed via telephone. Surveys were sent to households. | The first survey was administered September 1989. The study period lasted 88 weeks and had three follow-up mailings | Community water system using wells contaminated with Trichloroethylene. Some respondents reported hauling water as alternative | Drinking water (Household use such as cooking was implied) | Both qualitative and quantitative (i.e. Mail questionnaires were sent to Perkasie residents) |
| T. J. Centner | Oversight of shale gas production  in the United States and the disclosure of toxic substances | Journal Article | 2013 | Unites States - specifically 22 states with known shale gas drilling legal or regulatory citation for disclosure of chemicals. | National Level, seems to cover rural and urban | National (federal regulations) and state level (state regulations) though contamination is cited as a concern of individuals and communities. | Unspecified, study is a review of existing legislation before and up to 2013, water quality disclosure in 20+ states | Discusses both surface and ground water though it does not always specify if the health risk is only for one or the other. | Mentions drinking water but does not specify uses. Mentions use of water for fracking. | Qualitative review of existing laws and case studies. |
| D. H. Wrenn; H. A. Klaiber;  E. C. Jaenicke | Unconventional Shale Gas Development, Risk Perceptions, and Averting Behavior: Evidence from Bottled Water Purchases | Journal Article | 2016 | Pennsylvania as the treatment and Ohio as the control | Predominantly rural and mountainous shale counties | County level - looked at shale gas counties vs non shale gas counties. Treatment (PA) were paired with a control (OH). Assumption that a large portion of rural counties use private wells. | Uses home scan data from 2005-10 for bottled water purchases. | Wells | Household | Quantitative Looks at existing data |
| S. Gopalakrishnan;  H. A. Klaiber | Is the Shale Energy Boom a Bust  for Nearby Residents? Evidence from Housing Values in Pennsylvania | Journal Article | 2013 | Washington County, PA | Rural/suburban (Peri-urban). It is located outside of Pittsburgh | Housing data obtained for a final sample of 3646 single-family residential transactions.  Looking at household level data within a single county. | Researchers purchased data from Dataquick that was collected from Jan 2008 - Oct 2010. | Private drinking water wells for some homes. 91% of homes fell in water providers coverage areas. | Mentions well water for household use (i.e. drinking) also mentions some homes had pools though it is not clear which of these also had wells. | Analysis of qualitative and quantitative data. Qualitative from Dataquick, Qualitative from a LexisNexis search of newspapers |
| L. Merkel; C. Bicking;  D. Sekhar | Parents' Perceptions of Water  Safety and Quality | Journal Article | 2014 | Across PA | All | Full time caregivers of children with permanent residence in PA. | data collection via internet survey was completed in Spring of 2011 | well water (2.9% of respondents) | Household water uses (i.e. drinking). | Mixed: Survey included quantitative data as well as open ended responses. |
| L. Muehlenbachs;  E. Spiller; C. Timmins | The Housing Market Impacts of  Shale Gas Development | Journal Article | 2015 | 36 counties in PA | All | Differentiation between houses by proximity to shale gas wells and water source. | Real estate data was collected by CoreLogic from Jan 1995 to April 2012 Shale gas well drilling sites (& dates) were obtained from PADEP Spud data and DCNR well information system. | Private wells | household use (Also mentioned water was transported to and from shale gas wells for use and disposal) | quantitative |
| P. M. Rabinowitz;  I. B. Slizovskiy; V. Lamers; S. J. Trufan; T. R. Holford; J. D. Dziura; P. N. Peduzzi; M. J. Kane; J. S. Reif; T. R. Weiss; M. H. Stowe | Proximity to Natural Gas Wells and Reported Health Status: Results of a Household Survey in Washington County, Pennsylvania | Journal Article | 2015 | 38 rural townships within the center of Washington Co, PA. | Rural | household level - specifically has the respondent or anyone in the household experienced each medical condition. | Household survey was conducted in 2012. The spud (drilling date) of most of the wells fell between 2008 and 2012. (95%) | Ground fed well or spring. | drinking, cooking, showering, and swimming. | Mixed methods. Data was obtained and surveys were administered that collected qualitative and quantitative data. |
| B. R. Swistock; S. Clemens; W. E. Sharpe | Drinking water quality in rural pennsylvania and the effect of management practices | Legal Bried | 2009 | Private Wells across PA | rural | Household | 2006-2007: 700 private wells were sampled. In 2007, 450 participated in a follow up survey where data on concerns was collected. | Private Wells and Roadside Springs. All participants owned a private well though 9% chose to use springs for drinking water 8% part of the time and 1% all of the time. | household use | mixed method. Quantitative data as well as qualitative from the survey. |
| B. R. Swistock; J. Clark; S. Bosner; D. Oleson; A. Galford; G. Micsky; M. Madden | Issues Associated with the Use of Untreated Roadside Springs as a Source of Drinking Water | Journal Article | 2015 | roadside spring locations were chosen in 21 counties across PA, particularly Western and Northern PA | Rural (most roadside springs were found in rural areas) | Water samples taken from 37 roadside springs in 21 counties. 1035 attendees at 56 Penn State Extension workshops answered questions about roadside springs. | Roadside spring samples collected between April 2013 and May 2014, survey questions about use were asked in 2014 and 2015 | Roadside springs, which source water from shallow groundwater | drinking water, water plants | mixed methods |
